# Hazardous base surges of Taal’s 2020 eruption

**DOI:** 10.1038/s41598-021-94866-2

**Published:** 2021-08-03

**Authors:** A. M. F. Lagmay, M. I. R. Balangue-Tarriela, M. Aurelio, R. Ybanez, A. Bonus-Ybanez, J. Sulapas, C. Baldago, D. M. Sarmiento, H. Cabria, R. Rodolfo, D. J. Rafael, J. R. Trinidad, E. Obille, N. Rosell

**Affiliations:** 1 UP National Insitute of Geological Sciences, College of Science, University of the Philippines, Diliman, Quezon City, Philippines; 2 UP Resilience Institute and NOAH Center, University of the Philippines, Diliman, Quezon City, Metro Manila, Philippines; 3 Agriculture Sustainability Initiatives for Nature, Inc., Diliman, Quezon City, Philippines; 4 Department of Environmental Science, Ateneo De Manila University, Loyola heights, Quezon City, Metro Manila, Philippines; 5 National Institute for Science and Mathematics Education Development, University of the Philippines, Diliman, Quezon City, Philippines

**Keywords:** Natural hazards, Volcanology

## Abstract

After 43 years of repose, Taal Volcano erupted on 12 January 2020 forming hazardous base surges. Using field, remote sensing (i.e. UAV and LiDAR), and numerical methods, we gathered primary data to generate well-constrained observed information on dune bedform characteristics, impact dynamic pressures and velocities of base surges. This is to advance our knowledge on this type of hazard to understand and evaluate its consequences and risks. The dilute and wet surges traveled at 50-60 ms^−1^ near the crater rim and decelerated before making impact on coastal communities with dynamic pressures of at least 1.7 kPa. The base surges killed more than a thousand livestock in the southeast of Taal Volcano Island, and then traveled another ~ 600 m offshore. This work is a rare document of a complete, fresh, and practically undisturbed base surge deposit, important in the study of dune deposits formed by volcanic and other processes on Earth and other planets.

## Introduction

After 43 years of repose, Taal Volcano erupted on 12 January 2020 forming a 17–21-km high plume^1–3^ causing prolonged widespread disruption to the normal daily activities of surrounding populations. Only one death was directly attributed to the explosive eruption, the body of a person retrieved under a ~ 1.5 m-thick pyroclastic deposit on the west coast of Taal Volcano Island (TVI). Another man who failed to evacuate from the island is still missing^4^. Considering the sudden and explosive nature of Taal Volcano’s 2020 eruption that generated base surges, the number of recorded fatalities could have easily been higher.

Base surges are one of the most lethal and destructive hazards of Taal’s historical eruptions^5^. Taal Volcano’s 1965 eruption was well-documented by J.G. Moore^6,7^, who after observations of similar basal flows in nuclear blasts, coined the word “Base Surge”, a term still used in the volcanological literature and by warning agencies until today^8–10^. Base surges are the dilute, wet, and turbulent end-member of Pyroclastic Density Currents (PDCs), which are gravity-driven flows generated by the collapse and lateral spreading of hot gas particle-laden mixtures produced during explosive volcanic eruptions^11,12^. Base surges form when magma and water interact during explosive phreatomagmatic eruptions^10,13–18^.

The 1911 eruption of Taal Volcano killed 1,335 people^19^, whereas the 1965 eruption killed 200^6^. Other eruptive events, such as in AD1716, AD1731, AD1749, and AD1754, were also described to produce base surges resulting in thousands of deaths^20^. Out of the 34 recorded historical eruptions since AD1572, Taal had six distinct eruptions reported to have generated base surges^19,20^. Here, we report the seventh distinct eruption of Taal Volcano that generated base surges. Until the time of this writing, there is no verbal account nor published report on the occurrence of base surges from Taal Volcano’s 2020 eruption.

The 2020 base surges were accidentally discovered during an ecological expedition on the barren Taal Volcano Island (TVI) to search for signs of life (i.e. flora and fauna) and clues on how to make the area productive again^21^. These base surges were mapped in the field with the aid of satellite and drone technologies. Pre-eruption Light Detection and Ranging (LiDAR) data and post-eruption drone-generated Digital Terrain Models (DTMs) were used to measure the thickness and estimate the volume of the latest surge deposits of Taal Volcano. The dune field in the southeast sector offers a rare, largely undisturbed, and nearly complete picture of a base surge deposit field, which we describe concisely in this article. Often, these deposits are studied as discontinuous outcrops years after an eruptive event (e.g. Maungataketake, New Zealand; Tungurahua, Ecuador; Taal, Philippines), when erosion, deposition, and vegetation have obliterated or concealed important features that hinder better understanding of flow emplacement and dynamics^22–24^.

As one of the 16 Decade Volcanoes^25–27^ identified by the International Association of Volcanology and Chemistry of the Earth’s Interior (IAVCEI), this work on the Taal Volcano 2020 base surges is of particular importance because of the destructive nature and proximity of the volcano to densely populated areas. The results can also be used to compare dune deposits formed by volcanic, and other processes on Earth and other planets^28–30^.

## Results

### Analysis of time-series imagery and video analysis

**Figure 1:**
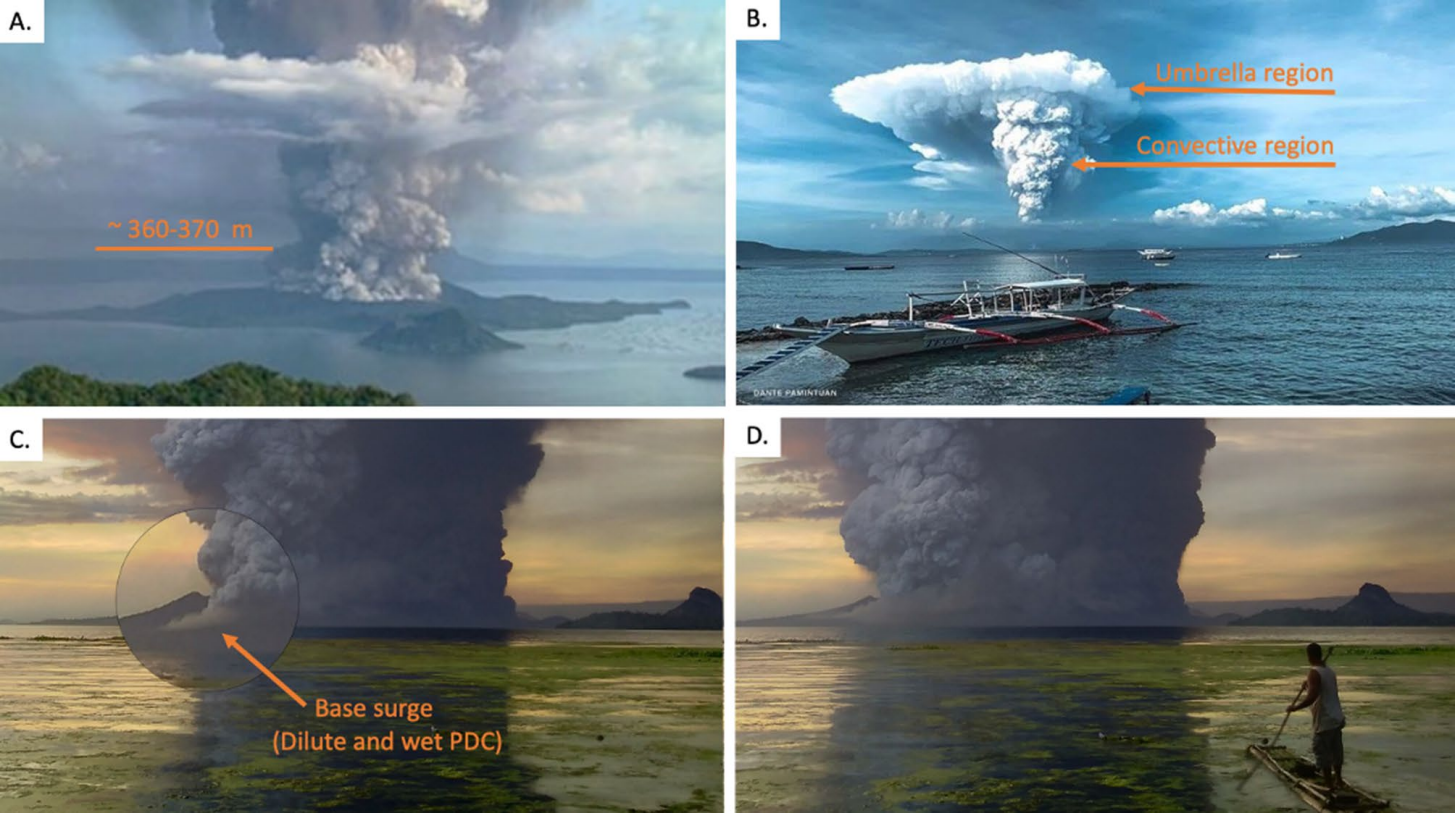
Photographs and screenshots of videos of the 12 January 2020 eruption of Taal. (**A**) Eruption showing a lateral basal cloud with an estimated fountain height of 360-370 m. Photo by Johnny Alegre (CC-BY SA4.0). (**B**) Umbrella cloud and convective regions. Photo by David Comes Lorenzo. (**C**) Magnified view of base surges (dilute and “wet” PDCs) forming in the southeast sector of TVI; (**D**) Dust covering the southeastern slopes of TVI. Photos C and D by Andres Alisuag.

The 12 January 2020 eruption of Taal Volcano generated a vertical volcanic eruption column consisting of a gas thrust (jet phase), a convective, and an umbrella region^31^. The gas thrust region rose to ~ 130 meters above the Main Crater (MC) rim and is identified by the region where ballistic volcanic bombs and laterally moving flows (basal clouds) traveling at 50-60 ms^−1^ are observed. In the convective region, ingested air continued to expand the plume reducing its density. This caused the discharging mixture of hot gas and pyroclasts to rise and form a gray-colored column of billowing clouds that thickened upward. By around 4:00 pm, a north-drifting umbrella cloud is well-formed (Fig. 1), which by 8:00 pm reached a height of 17-21 km^1–3^ with an E-W diameter of ~ 100 km. The eruption height and umbrella diameter suggest that the eruption was characterized by mass discharge rates^31,32^ in the order of 10^7^ kgs^−1^ equivalent to a Volcanic Explosivity Index (VEI)=4. Intense activity lasted up to about 10 hours and started to wane in the morning of 13 January 2020 at about 2:49 am when lava fountaining was observed^33^. Volcanic activity on 13 January 2020 was characterized by a series of discrete, cannon-like explosions^34^ that were directed towards the west (see supplementary video).

### Thickness and volume

Based on the analysis of pre- and post-2020 eruption DTMs of the southeast sector of TVI, the base surge deposits of Taal Volcano are thickest on the upper slopes (50–180 m elevation) where the gradient is on average about 17°. The maximum and average thickness on these slopes are 12 m and 4.7 m, respectively (Fig. 3). In the middle part of the southeast sector (20–80 m elevation), where the average slope gradient is 13°, the maximum thickness is 11 m whereas the average thickness is 2.6 m. The lower slopes near the coast (4–26 m elevation), with an average gradient of 8° have the thinnest deposits with a maximum and average thickness of 5.8 m and 0.9 m, respectively.

The base surge deposits drape differentially over undulating topography. Deposits are relatively thin at the crest of hills becoming thicker at their base. This is observed almost everywhere in the dune field except for an area where there is a conspicuous topographic bulge at the lower half of the upper-slope section (Fig. 3). Upon closer examination of topographic profiles, this sudden change in relief reflects the frontal edge of an older pre-2020 PDC deposit draped by new deposits. In this area, dilute PDC deposits are thick at the top of the old deposit and abruptly become thin downslope of the bulge. Sudden thinning of the deposit downstream of the bulge may be due to the pre-existing gully that channelled the base surge or air entrainment as the current jumped over the bulge favoring suspension. In other areas of the lower slopes, base surge deposits become thinner immediately upon crossing a pre-eruption river channel (Fig. 3). The deposit thickens after a few tens of meters and suddenly thins out again after another river channel is crossed in the dendritic drainage network of the southeast flank.

**Figure 2:**
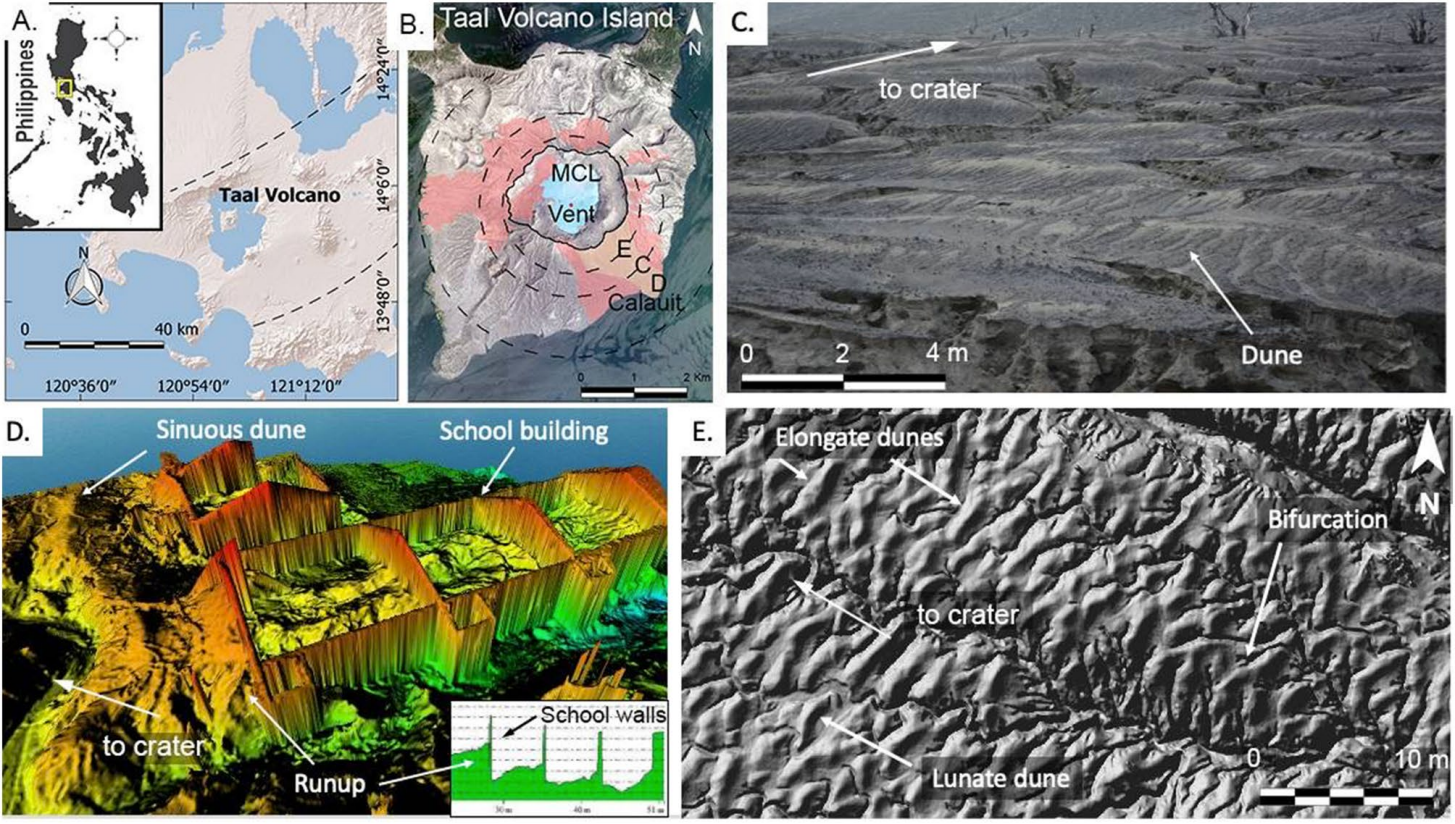
Dunes of TVI. (**A**) General location of Taal Volcano Island. The area within the dashed lines is the Macolod Corridor, a 40-km-wide rift zone^65^. (**B**) Distribution of the 2020 base surge dunes. Circles refer to upper slope, middle slope, and lower slope boundaries. Areas with dune forms identified through satellite imagery are in red. Field validation was done in the southeast flank (yellow portion of map). K3 and ASTI (“Philippine Copyright 2021 by DOST-ASTI”, includes material (c) KARI 2021, Distribution (SI Imaging Services, Republic of Korea), all rights reserved. (**C**) Photograph of the dune field. (**D**) Digital Surface Model of school overwhelmed by base surges. Sinuous dunes and runup of the base surge also occur inside the classrooms. (**E**) Plan view image of dunes showing elongate and lunate dunes. Bifurcation of dunes is also shown.

The estimated volume of the base surge deposits on TVI, which cover an area of 6.2 million m^2^ (Fig. 2), is 19 ± 3 million m^3^ with a Dense Rock Equivalent (DRE)^35^ of 10 ±1.6 million m^3^. The thickness of the deposit at the coast is 0.9 m on average, which indicates that the base surges reached the outer lake. Based on the extent of base surge dunes in the west, our calculations placed the end of the density current in the southeast sector ~ 600 m offshore the village of Calauit (see Fig. 2B).

### Morphology

Remotely sensed images show the entire TVI covered by light gray tephra. Close-up views reveal a distinct mottled texture that arises from dune-like forms, typical of base surge deposits^6,7^. The outlines of these dunes were delineated manually from the crater rim to the coast of the island on the southeastern and western slopes. They are also present in areas in the north but do not extend up to the coast of TVI (Fig. 2). There are no dunes in the southwest slopes because of the high elevation of the main crater rim in the southwest. The distribution suggests radial spreading of the base surges as a result of column collapse^7^ with possible contribution from discrete “cocktail jets”, “radially-overpressured jets” and “eruption slugs”^13–15,17,36^.

The southeast sector is characterized by a desert-like dune field (Fig. 2) and incised channels from surface water runoff erosion. The larger incised channels or gullies generally follow pre-eruption drainage whereas the smaller incised channels are mainly controlled by the dune relief. Larger dunes bear erosion marks that resemble a trellis drainage pattern that connect to smaller incised channels at the base of and in between dunes. These smaller incised channels, in turn, connect to larger gullies.

**Figure 3:**
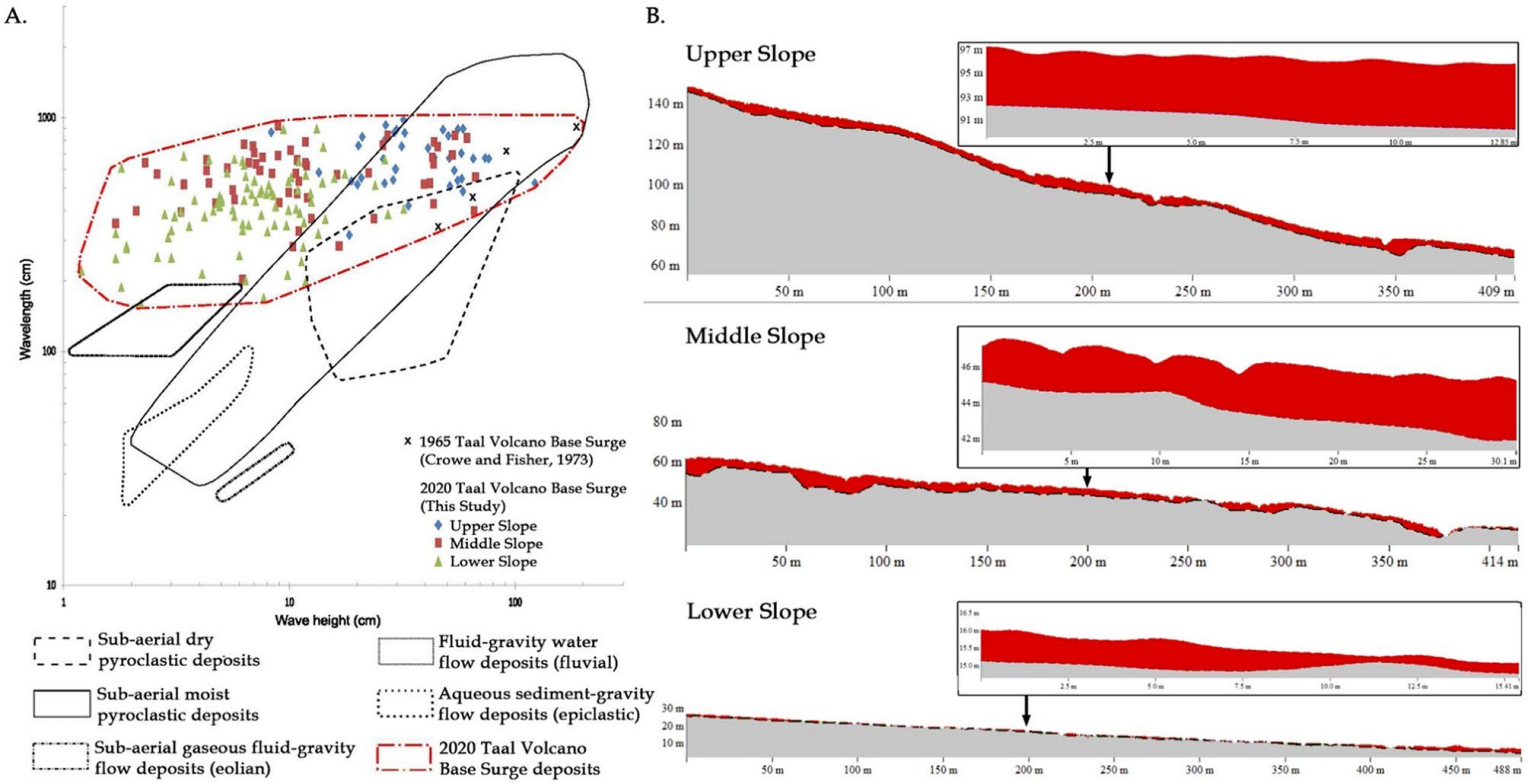
(**A**) Graph of dune waveheight vs wavelength for subaerial dry and moist pyroclastic flows, eolian, fluvial and aqueous sediment-gravity (epiclastic) flows. Modified from Moorehouse and White (2016) (**B**) Thickness of surge deposits in the upper, middle, and lower slopes of the southeast sector of TVI. Profiles also show dunes with stoss and lee sides at different gradients of underlying slope.

Base surge dunes in the eastern sector of TVI are generally oriented parallel to the southeast crater rim and are most prominent at mid-slopes. The ratio of dune wave height (0.12–0.80 m) and wavelength (1.1–9.0 m) scales down towards gentler slopes at low elevations (Fig. 3) with wave height decreasing progressively, which reflects waning of the density current. The ratios of wave height to wavelength plot partly within the field of base surges (moist PDCs) in pyroclastic dunes studied worldwide^37^. This field of moist PDCs in the diagram is hereby expanded because of the morphometry of dunes in the mid-slopes and those near the coast, which exhibit smaller wave height to wavelength ratios.

Dunes are asymmetrical with the stoss side invariably shorter than the lee side (Fig. 3). When measured relative to the horizontal plane, the stoss sides of dunes have gentler slopes compared to their lee sides along steep slopes of the volcano. Nearer to the coast where the volcano gradient is nearly flat, the stoss side is steeper than the lee side. However, when stoss and lee angles are measured relative to the dip angle of the underlying slope, steepness of the stoss is always higher than the lee side of dunes. This observation where the underlying topography affects the measurement angle of the stoss and lee sides has implications to the study of outcrops of old dune formations produced by dilute PDCs. When not recognized, this may lead to difficulties in the interpretation of flow direction and formation mechanisms of bedforms^22,24,38^.

Dune bedforms are mostly elongated with lengths ranging from 3.9 - 12.6 m and are sinuous or lunate in planform. The bedforms have a mean length of 5.7 m with a standard deviation of 1.9 m. Lunate dunes^22^ are dominantly crescent-shaped and concave downstream. They are commonly found at the lower slopes whereas sinuous dunes are more abundant at the middle and upper slopes. Occasional bifurcation of dunes was also observed. Dune bedforms are not readily apparent in the area a few tens of meters from the crater rim but closer examination in the field reveals the presence of slight bulges on the surface with underlying cross-bedded structures.

### Stratigraphy and componentry

The stratigraphy of the 2020 base surge deposits in southeast TVI is composed of alternating undulating beds and laminae which are, in general, relatively coarser near the crater compared to those near the coast (Fig. 5). This suggests that the density currents were losing energy and carrying capacity as they traveled downslope. When foresets and backsets of beds and laminae of non-uniform thickness connect, they form dunes. Laterally continuous, horizontal planar beds with equal thickness and well-sorted components are less pervasive. When present, they may indicate the contribution from fall out of pyroclasts. The presence of accretionary lapili in almost all of the deposits, plastered deposits on vertical walls, as well as abundance of juveniles and accidental clasts, constitute strong evidence that suggests a wet density current formed from water-magma interaction^10^.

**Figure 4:**
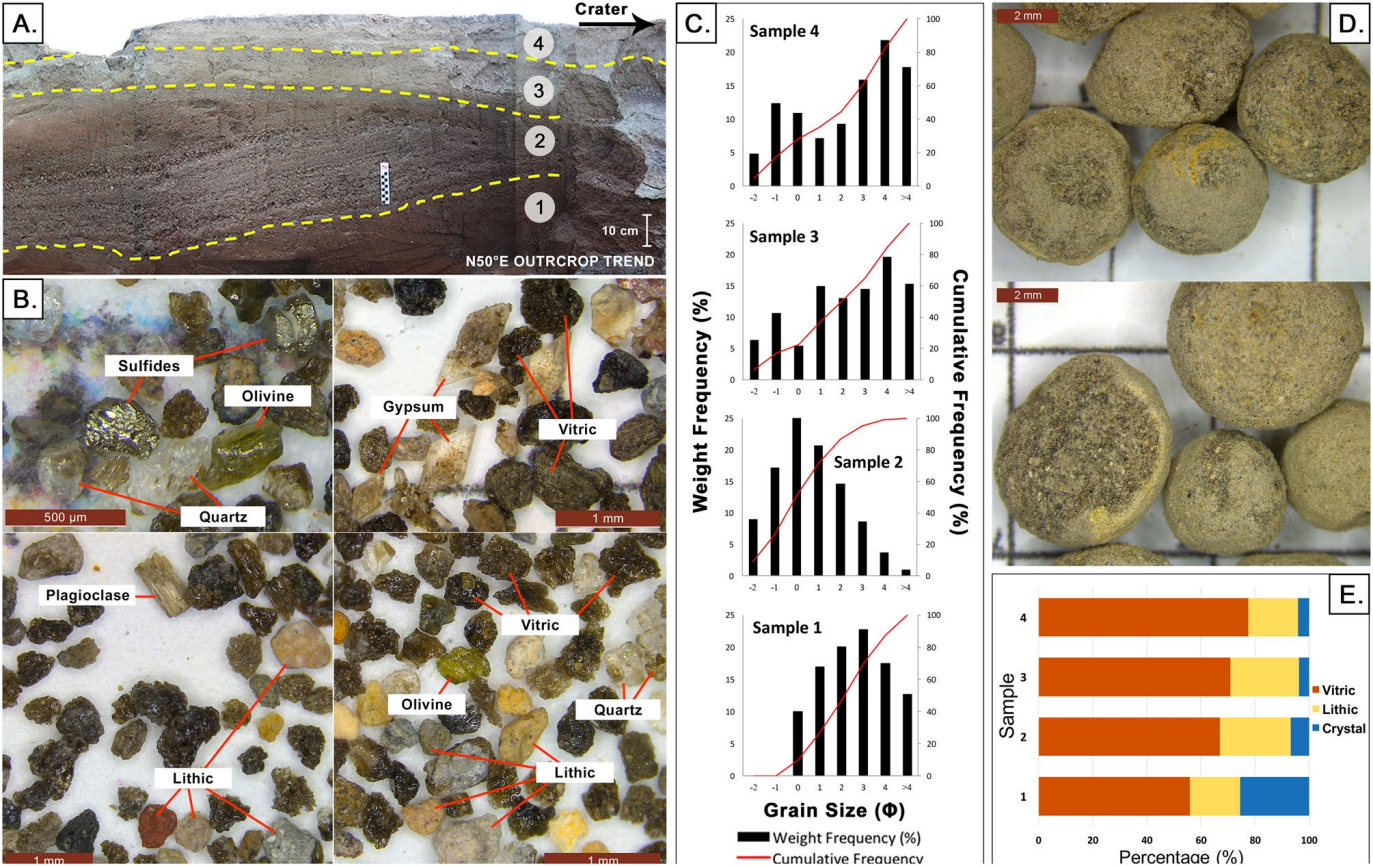
(**A**) Station 1 (14.002304°, 121.008571° Located at Fig. 5D) where samples were collected; (**B**) Ash components including vitric, lithic, and crystals (gypsum, olivine, plagioclase, quartz crystals), as well as secondary quartz and sulfide fragments from the mineralized hydrothermal system; (**C**) Grain size distribution graph per sampled layer; (**D**) Accretionary lapilli found in Samples 3 and 4; (**E**) Relative abundance of the components of ash per sample.

The pre-2020 eruption topography is marked by the presence of plant debris. Overlying the unconformity is a poorly sorted 0.2-m bedded tephra deposit dominated by ash-sized pyroclasts. This bed is overlain by cross-bedded deposits dominated by lapilli-sized lithic and crystal fragments. Some of the tephra layers show grading, both normal and reverse. The number of irregularly stacked dunes is variable, ranging from 1-4 dune bed forms of variable sizes with outcrops in the mid-slope having the most number in a stack. In between stacked dunes are differential draping beds that fill the trough between laterally adjacent dunes. Laminae composed of poorly-sorted finer-grained tephra cap the dunes. Overall, there is an increase in the proportion of fine components in strata towards the top of each outcrop. These stratified beds and laminae of the surge deposits are believed by many to have formed from numerous explosions that generate particle-laden density currents that vary in velocity during transport^38–42^. Lastly, the size-frequency distribution in all sampled beds indicates poor sorting reflective of deposition from a density current. Some beds exhibit a weakly bimodal distribution (Figure 4).

**Figure 5:**
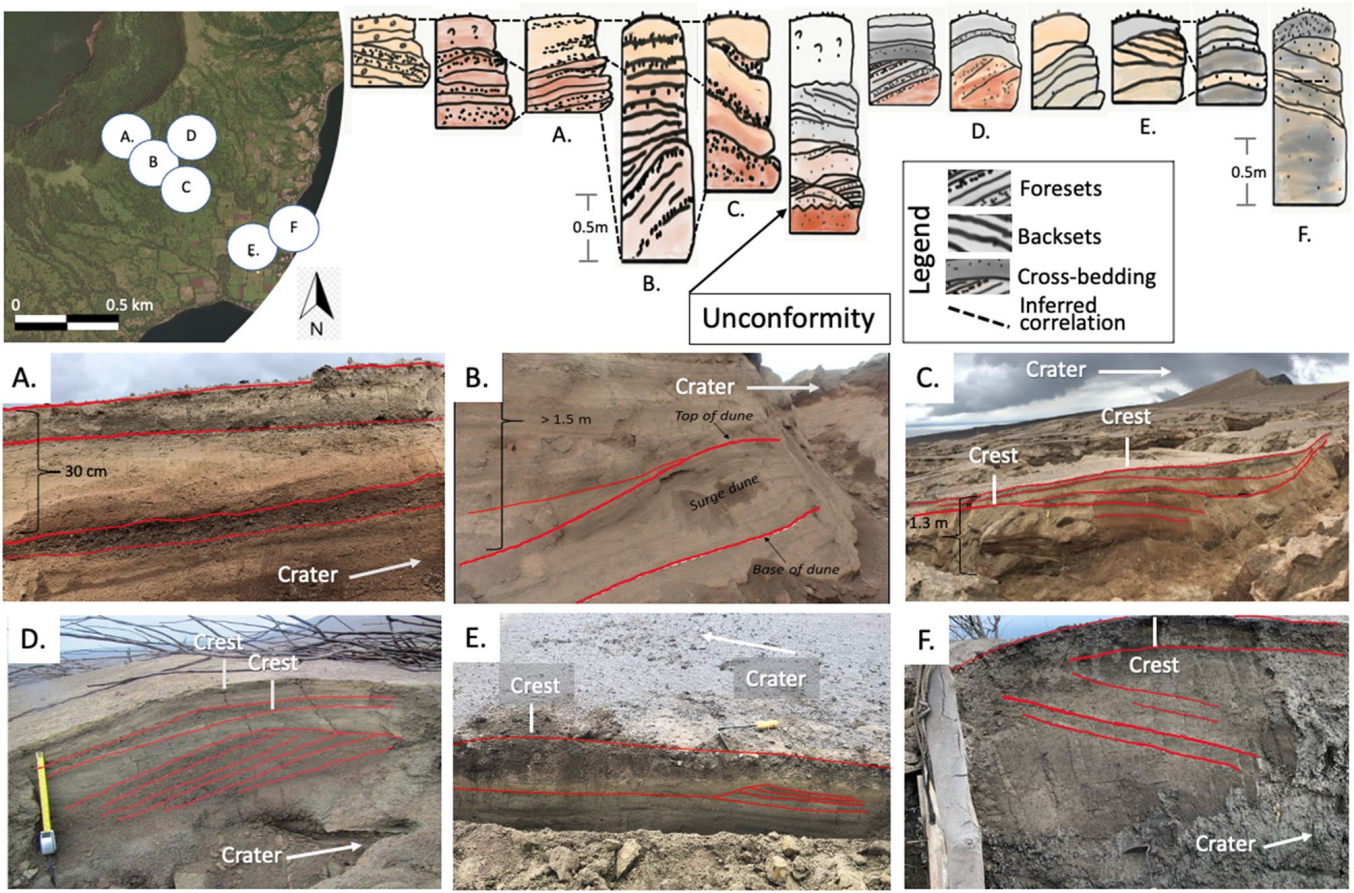
Stratigraphy of different dune bedforms in the southeast sector of TVI showing foresets, backsets, erosional features, curved surfaces, and ballistic fragments on the surface. See locations designated by letters** A**–**F**.

Nearly all sections of dunes have cross-bedded structures with dipping planar beds inclined by about 5°–18° (Fig. 5). Foresets and backsets in many sections of dunes are typically truncated by overlying strata and are interpreted as limbs of earlier-formed dunes that were eroded at their crests by succeeding flows. In the lower slopes, backsets are steeper than foresets whereas in the proximal to medial areas where the underlying slopes are steeper, the opposite is observed (see discussion on stoss and lee angles above). There is aggradation or regression in the upstream direction and migration of the crest of stacked dunes towards the crater typical of antidunes (Fig. 5C,E,F). However, there were two adjacent outcrops beside a gully where migration of the crest of stacked dunes is toward the downstream direction (Fig. 5D).

Volcanic glass (vitric), lithics, and crystal fragments comprise the base surge beds (Fig. 4C). The vitric component is commonly light brown to black, translucent, and exhibits blocky, scoriaceous, and fluted forms. Bubble wall shards with varying vesicularity were observed in some fractions but with dominance of blocky vitric components suggestive of a phreatomagmatic eruption origin^42^. Lithic fragments plucked from the wall rock or conduit, include sub-rounded rock fragments, oxidized grains, as well as hydrothermal fragments such as those observed in hydrothermal ore deposits (white to yellow in appearance). Pyrogenic crystal components (e.g. gypsum, olivine, plagioclase, quartz) consist of euhedral to fragmented free crystals, with some still embedded in glassy groundmass, while hydrothermal minerals (e.g. sulfides, quartz) were mostly observed with the hydrothermal lithics. All samples contain the 3 tephra components, but are generally dominated by volcanic glass (56%-77%) with varying morphologies. Lithics are composed of 18–26% hydrothermal fragments (e.g altered volcanic lithics) with an insignificant amount of rock grains. Crystal fragments have the lowest portion in all the layers (< 25%).

### Impacts

**Figure 6:**
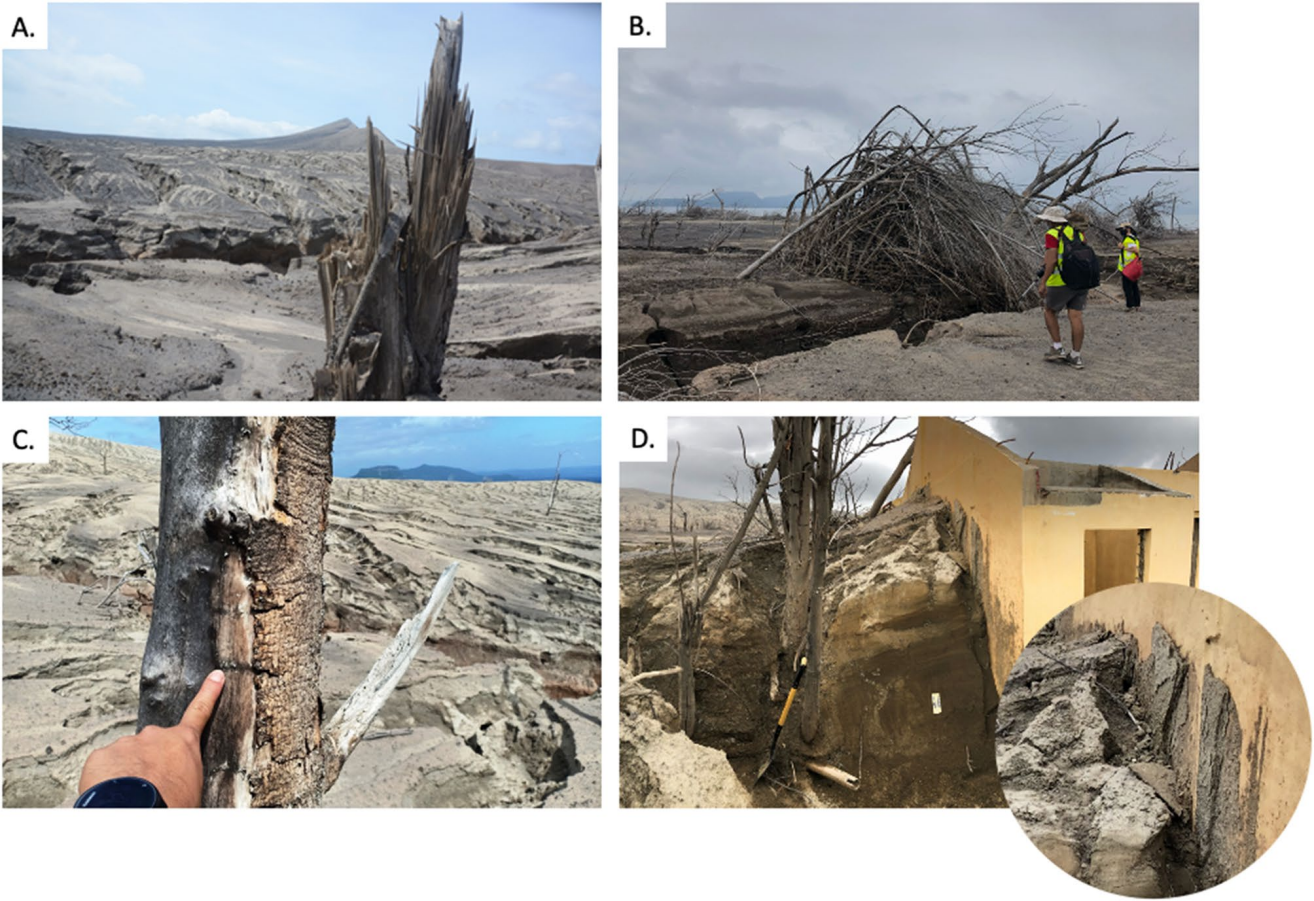
Photos of the effect of base surges on the southeast portion of TVI. (**A**) Snapped and splintered tree trunk of *Ceiba pentrada* with trunk diameter ~ 0.5 m; (**B**) Ruptured bamboo (Culm diameter = 12 cm); (**C**) Scorched and debarked tree on the side facing the crater; (**D**) Base surge deposit run-up against school wall facing the crater. Inset photo shows plastered base surge deposits. This unique feature of pasty materials persistently sticking on walls is proposed to be called “ludite”.

Previously vegetated areas in the southeast sector of TVI were reduced to a desert dune field with fallen trees, ruptured bamboo, and splintered tree trunks (Fig. 6A,B). The unidirectional blowdown was pronounced near large gullies where base surges were funneled. Trees were debarked and sandblasted mainly on the side facing the main explosion crater with some scorched but not completely turned into charcoal (Fig. 6C). This suggests that these wet surges were at a minimum temperature of 200° C^43,44^. In this part of the island, regrowth of trees a year after the 2020 eruption is not evident.

Velocity of the base surges near the crater rim is estimated to be ~ 50–60 ms^−1^ based on video analysis. When considering the maximum solution with a 16% exceedance probability as a safety value^45,46^, calculated dynamic pressures using PYFLOW_2.0 at the upper slopes (1.3 km from the vent) reflect values typical of a dilute PDC that can cause light to moderate building damage^47^, with values ranging from 3.5 kPa over the first 2.5 m (typical height of a 1-storey house) to 5.2 kPa over the deposit height. The calculated velocities at this location show a maximum of 48 ms^−1^ over the deposit height, increasing to 57 ms^−1^ over a height of 2.5 m.

Downstream, a splintered *Ceiba pentrada*^48^ in the lower mid-slopes (Fig. 6A) and a ruptured bamboo located beside a gully (Fig. 6B) indicate dynamic pressures in excess of 2.1 ± 0.6 kPa and 1.7 ±0.5 kPa^44,49^, respectively. Their equivalent velocities are 40 ± 6 ms^−1^ and 36 ± 6 ms^−1^ using a minimum shear flow density of 2.7 kgm^−3^. Further downslope, PYFLOW_2.0 estimated a maximum velocity of 14 ms^−1^ near the coast (2.2 km from the vent). The computed velocities show a decreasing trend from the crater to the coast. These velocities are consistent with estimates using the energy-line method^50–52^ and show deceleration despite a concave upward slope profile^53^ of Taal Volcano.

Buildings along the coast had collapsed roofs made of galvanized iron sheets and were buried by about 1-2 m of base surge deposits. Some areas were plastered by vertically-oriented and bedded muddy coating (Fig. 6D). Scattered ballistic projectiles, mainly composed of scoria bombs and minor altered lithic fragments, are found on the surface of the surge deposit field. They range from lapilli- to block-sized clasts with scoria bombs decreasing in size towards the coast. Multiple base surge flows impacted the community based on evidence of stacked dune bedforms. Temperatures were also significantly high to scorch trees in the community and can be lethal. Lastly, even at low temperatures and dynamic pressures, prolonged exposure to inhalable hot fine ash reduces the chance of survival^54^.

## Discussion

Base surges are considered as a main hazard of Taal Volcano. Mobile and water vapor-rich, they can travel at velocities greater than 30 ms^−1^ and bury everything in their path^7,8^. This work provides primary data and observed information, useful to advance our understanding of base surges and evaluating consequences and risks of such eruptions^17,55^.

The 17–21 km-high phreatomagmatic eruption of Taal in 2020 has equivalent mass discharge rates in the order of 10^7^ kgs^−1^ with VEI=4. Base surges spread radially on the island from fountain collapse heights^31^ of ~ 360-370 m based on detailed analysis of photographs. A total base surge volume of 19 ±3 million m^3^ was deposited on the island beginning late afternoon, which most likely continued for another 10 hours based on peak activity as reviewed from official bulletins and seismic records. Topographic lows in the southeast and west of the MC rim allowed base surges to reach coastal areas extending ~ 600 m offshore the village of Calauit in the southeast sector of TVI.

A total of 480 families in Calauit were lucky to have evacuated the village hours before it was completely overwhelmed by lethal and destructive base surges. Unfortunately there was not enough lead time to evacuate 480 cattle, 270 horses, 70 carabaos, 276 goats, and more than a thousand swine and poultry^56^. All were declared in the livestock mortality report submitted to the Department of Agriculture.

Dunes characterize the base surge deposits on TVI. These elongated and sinuous mounds are perpendicular to sub-perpendicular to the direction of the density current as checked against the orientation of the blowdown of trees. Wave height to wavelength ratios of dunes partially plot in the moist PDC field of those studied worldwide. The unique morphometry of dunes in the mid-slopes and those nearer the coast, exhibiting smaller wave height to wavelength ratios, extends the field of moist PDCs. Dune profiles exhibit both progressive and regressive migration of dune crests, eroded foresets and backsets, cross stratification, pinch and swell draping, and other sedimentary structures that provide a rich source of information that can contribute to the discussions on the flow regime and emplacement of pyroclastic density currents based on bedform characteristics^22–24,39,44,57–60^.

**Figure 7:**
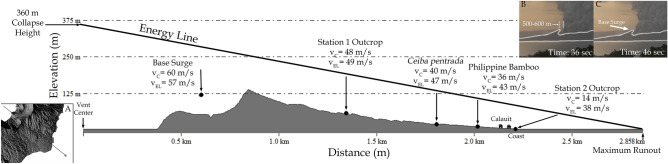
Projection of energy line model along the southeast profile of Taal Volcano Island where observation and calculation points for velocity are located from the vent center to the projected maximum runout distance. Velocities calculated using different methods (vC) and velocities calculated using the energy line model (vEL) for each of the four observation points are shown. (**A**) Location of southeast profile and observation points. (**B**) Image from timelapse video at 36 seconds showing 500–600 m open area within the southeast inner crater. (**C**) Image from time-lapse video at 46 seconds showing the area from B being covered by a moving base surge.

Dynamic pressure and velocity estimates based on video analysis, numerical simulations, and impact calculations for broken and splintered *Ceiba pentrada*^48^ and ruptured bamboo, show a decreasing trend from 50–60 ms^−1^ from the crater to 48 ms^−1^ in the upper middle slopes to 36–40 ms^−1^ near the gullies of lower slopes to 14 ms^−1^ near the coast. Structural impacts of these dynamic pressures on the village of Calauit were enough to destroy windows but not topple the walls of 1-storey buildings made of reinforced (steel bars) concrete walls. The sequence of deposits suggests that the roof of houses collapsed due to tephra accumulation prior to the arrival of base surges. Lastly, computed velocities are generally consistent with estimates using the energy line model^50–52^ and the base surges decelerated from the crater rim to the coast (Figure 7). This is inconsistent with the models expected for a volcano with a concave upward slope profile like Taal Volcano, where acceleration is first to be expected before deceleration^53^.

## Methodology

### Review of images and videos

Hundreds of photos taken by residents around Taal Lake, weekend tourists and passengers of commercial airplanes were reviewed to determine the type of eruption that took place on 12 January 2020 and 13 January 2020. Time-lapse videos taken by the authors on 13 January from Tagaytay, north of TVI, were also reviewed. Analysis of the photos include the delineation of low areas of the MCL crater rim and possible overflow by PDCs above the rim. The fountain collapse height, $H_{f}$, was measured from photographs to be around 130 m high from the eastern ridge of the TVI. We added the 235-m depth of the crater floor, which is 4 m above sea level, to come up with an $H_{f}$ range of 360–370 m that accounts for inaccuracies in estimation. This estimate is consistent with the expected range of fountain collapse heights according to Sparks (1997)^31^ given a discharge rate that corresponds to the 17–21 km plume height^1–3^ of Taal Volcano’s 2020 eruption.

Available LiDAR DTMs for the whole island was used to delineate the pre-2020 topography of TVI^61^ and for the thickness analysis of newly-formed base surge deposits. The DTMs were also used to measure the dune dimensions.

### Field work and sample collection

Field surveys were conducted on January 30 and February 13, 2021, a little over a year after the eruption of Taal Volcano. Targeted areas in the 2-day fieldwork of the southeast portion of TVI were based on the analysis of photographs and remotely sensed imagery. Tephra was sampled in-situ in different areas of the survey site using a container with fixed volume. These samples were then weighed in the laboratory for calculation of the bulk density of the base surge deposit, which was in turn used for the conversion of the base surge tephra volume estimate to DRE volume using the following equation:

1 $$\begin{aligned} DRE = \frac{Tephra\,Volume * Tephra\,Density}{Magma\,Density} \end{aligned}$$

where magma density used is 2700 kgm^−3^ (basaltic andesite magma), and tephra density is 1,425 kgm^−3^.

Descriptions of the tephra stratigraphy were conducted along exposures in rills ($\le$ 1 m deep) and gullies (3–7 m deep) at the southeast flank of the volcano. Sections were scraped to expose the stratigraphy. Dunes were also scraped perpendicular to the elongation axis of mounds to expose internal structures.

### Grain size and componentry analysis

At least 500 g to 1 kg of samples were collected per layer in the field to determine the size distribution of ash from individual layers. Grain size analyses were conducted with manual sieving. Cone and quartered samples were dried overnight at 60C to eliminate moisture. Sieving was performed using U.S.A. Standard Test Sieves ASTM E-11-20^62^ in the National Institute of Geological Sciences, University of the Philippines. Lithic, crystal, and vitric components were identified and described using a stereomicroscope, counting at least 1200 grains per sample.

### DEMs from Drones

A DJI Mavic Pro unmanned-aerial-vehicle (UAV) was used to obtain aerial imagery of parts of the southeastern sector of TVI. Selected regions of interest in the southeast sector were pre-programmed for mission flight paths covering areas of interest such as drainage, base surge dunes, and communities believed to have been ovewhelmed by dilute PDCs. Sequential photos along the flight path were collected with 60% overlap to generate orthomosaic images, point-clouds, and DSMs.

### Estimates of dynamic pressure and velocities

Dynamic pressures for the broken and splintered *Ceiba pentrada* (*r* = 25 cm, $\sigma _{ult}$ = 29,600 kPa^48^, $h_o$ = 25 m) and the ruptured bamboo (*r* = 6 cm, $\sigma _{ult}$ = 150,600 kPa^63^, $h_o$ = 15 m) were calculated using the following^49^:

2 $$\begin{aligned} P_{dyn}= \frac{1}{4}\frac{\pi r^2 \sigma _{ult}}{C_D h_o^2} \end{aligned}$$

where $P_{dyn}$ is the dynamic pressure, *r* is the radius of the tree, $\sigma _{ult}$ is the yield strength, $C_D$ (1.1) is the coefficient of drag, and $h_o$ is the estimated height of the trees.

Probability density functions of flow properties and impact parameters (e.g. velocity, density, dynamic pressure, rate and time of deposition) were solved based on the grain size and componentry analysis at two of the sampled outcrops (Station 1: 14.002304°, 121.008571° and Station 2: 13.99353°, 121.0119°) using PYFLOW_2.0^64^. The program reports the average (corresponding to 50th percentile), minimum (16th percentile), and maximum (84th percentile) solutions of each fluid dynamic variable. Values reported at specific heights are the maximum solutions with a 16% exceedance probability, considered here as a “maximum safety” value^45,46^. Using the dynamic pressures previously calculated and a minimum shear flow density of 2.7 kgm^−3^ computed by PYFLOW_2.0 at Station 2, the flow velocities at the location of the *Ceiba pentrada* and the ruptured bamboo were calculated with:

3 $$\begin{aligned} P_{dyn}= \frac{1}{2}{\rho }{v^2} \end{aligned}$$

where $\rho$ is the density and *v* is the velocity.

### Energy-line velocities

The mobility ratio (H/L) was calculated based on the observed collapse height (H = 360 m) from the time-lapse video whereas the run-out distance (L = 2,858 m) was measured from the center of the vent to the farthest extent of the dunes observed towards the coast. The resulting mobility ratio was plotted on a log-normal relationship showing the PDC‘s volume and mobility with 95% confidence and prediction limits^50^.

The maximum potential velocity of PDCs (Eq. 4)^50–52^ was derived based on the projected energy line model from the eruptive center of TVI.

4 $$\begin{aligned} v=(2g\Delta h)^{\frac{1}{2}} \end{aligned}$$

where *v* is the velocity, *g* is the acceleration due to gravity and *h* is the vertical distance between energy line and the ground surface. Points where velocity was observed or calculated using other methods were then plotted along the energy line profile to compare the resulting potential velocities from the energy line model (Fig. 7).

### Limitations

Only deposits in the southeastern areas of TVI are described in this paper with what can be accomplished in the span of two days of fieldwork with Alert Level 1 (Abnormal) hoisted over the entire Taal Volcano Island and Covid-19 modified quarantine restrictions still in place.

## Supplementary information

Supplementary material 1 (pdf 27 KB)

Supplementary material 2 (mov 22355 KB)

## Data Availability

The datasets used and analysed in this study are available on Google Drive at https://tinyurl.com/z74usc32. Use of the datasets can be cited as follows: “Lagmay et al., 2021 (this paper)”. The pre-eruption LiDAR DEM is openly available on DOST-Project NOAH’s Phil-LiDAR online portal: https://phillidar-dad.github.io/taal-open-lidar.html.

## References

[1] PTCC. Are you affected? (1991). https://fb.watch/4mw1qqclqI/.

[2] Perttu A (2020). Estimates of plume height from infrasound for regional volcano monitoring. J. Volcanol. Geotherm. Res..

[3] Bachmeier, S. Eruption of the Taal Volcano in the Philippines CIMSS Satellite Blog (2020). https://cimss.ssec.wisc.edu/satellite-blog/archives/35406.

[4] Cinco, M. Man found dead on Taal’s volcano Island (2020). https://newsinfo.inquirer.net/1229252/man-found-dead-on-taals-volcano-island.

[5] PHIVOLCS. Taal Volcano profile, In: Compilation of Eruptive History and Geology of Taal Volcano. Technical Report, PHIVOLCS-Library (1991).

[6] Moore JG, Nakamura K, Alcaraz A (1966). The 1965 eruption of taal volcano. Science.

[7] Moore J (1967). Base surge in recent volcanic eruptions. Bull. Volcanol..

[8] Hickson CJ (2013). Base Surge.

[9] PHIVOLCS. Summary of barangays susceptible to volcano base surge (2020). www.phivolcs.dost.gov.ph/vault/pdf/News/2020/BarangaysAtRiskTaal.BaseSurge-Buffer.pdf.

[10] Nemeth K, Kosik S (2020). Review of explosive hydrovolcanism. Geosciences.

[11] Druitt TH (1998). Pyroclastic density currents. Geol. Soc..

[12] Roche, O., Philips, J. & Karim, K. Pyroclastic Density Currents. In Fagents, S., Gregg, T. K. & Lopes, R. M. (eds.) Modeling Volcanic Processes (The Physics and Mathematics of Volcanism), chap. 10, 203–229 (Cambridge University Press, Cambridge England, 2013).

[13] Mastin L, Witter J (2000). The hazards of eruptions through lakes and seawater. J. Volcanol. Geotherm. Res..

[14] Belousov A, Belousova M (2001). Eruptive processes effects and deposits of the 1966 and the ancient basaltic phreatomagmatic eruptions in karymskoye lake, kamchatka, russia. Spec. Publ. Int. Assoc. Sedimentol..

[15] Nemeth K, Cronin S, Charley D, Harrison M, Garae E (2007). Exploding lakes in Vanuatu; “Surtseyan-style” eruptions witnesed on Ambae Island. Episodes.

[16] Dellino P, Isaia R, La Volpe L, Orsi G (2004). Interaction between particles transported by fallout and surge in the deposits of the Agnano-Monte Spina eruption (Campi Flegrei, Southern Italy). J. Volcanol. Geoth. Res..

[17] Brand B (2014). A combined field and numerical approach to understanding dilute pyroclastic density current dynamics and hazard potential: Auckland volcanic field, new zealand. J. Volcanol. Geotherm. Res..

[18] Dufek, J., Esposti, O. T. & Roche, O. Chapter 35-pyroclastic density currents: processes and models. In Sigurdsson, H. (ed.) *The Encyclopedia of Volcanoes*, 2nd edn, 617–629 (Academic Press, 2015). https://www.sciencedirect.com/science/article/pii/B9780123859389000353.

[19] Saderra, M. M. *The Eruption of Taal Volcano*. Bureau of Printing Manila Philippines (1911).

[20] Delos RPJ (2018). A synthesis and review of historical eruptions at Taal Volcano, Southern Luzon, Philippines. Earth Sci. Rev..

[21] Stoye, E. Tardigrade circus and a tree of life-January’s best science images. *Nature* (2021). https://www.nature.com/immersive/d41586-021-00095-y/index.html. 10.1038/d41586-021-00095-y 33547442

[22] Douillet GA (2013). Dune bedforms produced by dilute pyroclastic density currents from the august 2006 eruption of tungurahua volcano, ecuador. Bull. Volcanol..

[23] Douillet GA (2019). Pyroclastic dune bedforms: macroscale structures and lateral variations examples from the 2006 pyroclastic currents at tungurahua (ecuador). Sedimentology.

[24] Smith G (2020). A bedform phase diagram for dense granular currents. Nat. Commun..

[25] Barberi F (1990). (1990) Reducing volcanic disasters in the 1990’s. Bull. Volcanol. Soc. Japan.

[26] Torres R, Punongbayan R (1995). Attention focuses on Taal: decade volcano of the Philippines. EOS.

[27] Cas R (2019). IAVCEI: from small beginnings to a Vibrant International Association. Hist. Geo Space Sci..

[28] Thomas PC (1999). Bright dunes on Mars. Nature.

[29] Jackson DW, Bourke MC, Smyth TA (2015). The dune effect on sand-transporting winds on Mars. Nat. Commun..

[30] Smith KT (2018). Ghost dunes spotted on mars. Science.

[31] Sparks R (1997). Volcanic Plumes.

[32] Constantinescu R (2021). The radius of the umbrella cloud helps characterize large explosive volcanic eruptions. Commun. Earth Environ..

[33] PHIVOLCS. Taal Volcano eruption update 13 January 2020 04:00 PM (2020). https://www.phivolcs.dost.gov.ph/index.php/taal-volcano-bulletin-menu/9633-eruption-update-for-taal-volcano-13-january-2020-04-00-pm.

[34] Self S, WIlson L, Nairn IA (1979). Vulcanian eruption mechanisms. Nature.

[35] Crosweller HS (2012). Global database on large magnitude explosive volcanic eruptions (lameve). J. Appl. Volcanol..

[36] Dellino P (2010). Conduit flow experiments help constraining the regime of explosive eruptions. J. Geophys. Res. Solid Earth.

[37] Moorhouse BL, White JD (2016). Interpreting ambiguous bedforms to distinguish subaerial base surge from subaqueous density current deposits. Depos. Rec..

[38] Cole P (1991). Migration direction of sand-wave structures in pyroclastic-surge deposits: implications for depositional processes. Geology.

[39] Fisher RV, Waters AC (1970). Base surge bed forms in Maar volcanoes. Am. J. Sci..

[40] Schminke H-U, Fisher RV, Waters AC (1973). Antidune and chute and pool structures in the base surge deposits of the Laacher See area. Germany. Sedimentology.

[41] Sigurdsson H, Carey S, Fisher R (1987). The 1982 eruptions of El Chichon Volcano, Mexico: the physical properties of pyroclastic surges. Bull. Volcanol..

[42] Cas R, Wright J (1987). Volcanic Successions: Modern and Ancient.

[43] Browne, F. Theories on the combustion of wood. Technical Report, Madison, Wis.: U.S. Department of Agriculture, Forest Service, Forest Products Laboratory (1958).

[44] Brand B, Clarke A (2012). An unusually energetic basaltic phreatomagmatic eruption: using deposit characteristics to constrain dilute pyroclastic density current dynamics. J. Volcanol. Geoth. Res..

[45] Dellino P, Mele D, Sulpizio R, La Volpe L, Braia G (2008). A method for the calculation of the impact parameters of dilute pyroclastic density currents based on deposit particle characteristics. J. Geophys. Res. Solid Earth.

[46] Mele D (2015). Hazard of pyroclastic density currents at the Campi Flegrei Caldera (Southern Italy) as deduced from the combined use of facies architecture, physical modeling and statistics of the impact parameters. J. Volcanol. Geoth. Res..

[47] Baxter PJ (2005). The impacts of pyroclastic surges on buildings at the eruption of the soufriere hills volcano, montserrat. Bull. Volcanol..

[48] Green D, Winandy J, Kretschmann D (1999). Wood Handbook-Wood as an Engineering Material.

[49] Clarke A, Voight B (2000). Pyroclastic current dynamic pressure from aerodynamics of tree or pole blow-down. J. Volcanol. Geoth. Res..

[50] Toyos G, Cole PD, Felpeto A, Marti J (2007). A GIS-based methodology for hazard mapping of small volume pyroclastic density currents. Nat. Hazards.

[51] Tierz P (2016). Suitability of energy cone for probabilistic volcanic hazard assessment: validation tests at somma-vesuvius and campi flegrei (italy). Bull. Volcanol..

[52] Tierz P (2016). Uncertainty assessment of pyroclastic density currents at mount vesuvius (italy) simulated through the energy cone model. Nat. Hazard Uncertain. Assess..

[53] Valentine G, Doronzo D, Dellino P, de Tullio M (2011). Effects of volcano profile on dilute pyroclastic density currents: numerical simulation. Geology.

[54] Dellino P, Dioguardi F, Isaia R, Sulpizio R, Mele D (2021). The impact of pyroclastic density currents duration on humans: the case of the AD 79 eruption of Vesuvius. Sci. Rep..

[55] Cole, P. D., Neri, A. & Baxter, P. J. Hazards from pyroclastic density currents. In *The Encyclopedia of Volcanoes*, 943–956 (Elsevier, 2015).

[56] Robles, R. & Robles, T. Initial Visit to Jowivil Crater View Subdivision Balete, Batangas. Technical Report, First Asia Institute of Technology and Humanities (2020).

[57] Fisher R (1969). Bed forms in base-surge deposits: Lunar implications. Am. J. Sci..

[58] Waters AC, Fisher RV (1971). Base surges and their deposits: Capelinhos and taal volcanoes. J. Geophys. Res..

[59] Crowe BM, Fisher RV (1973). Sedimentary structures in base-surge deposits with special reference to cross-bedding, Ubehebe Craters, Death Valley, California. Geol. Soc. Am. Bull..

[60] Cole P, Scarpati C (1993). A facies interpretation of the eruption and emplacement mechanisms of the upper part of the Neapolitan Yellow Tuff, Campi Flegrei, Southern Italy. Bull. Volcanol..

[61] UP TCAGP & PHIL-LiDAR Program. LiDAR map of Taal Volcano (2020). https://phillidar-dad.github.io/taal-open-lidar.html.

[62] International ASTM. Standard Specification forWoven wire Test Sieve Cloth and Test Sieves. Technical Report, ASTM International, West Conshocken (2014).

[63] Bamboo Import. Mechanical Properties of Bamboo (2021). https://www.bambooimport.com/en/blog/what-are-the-mechanical-properties-of-bamboo.

[64] Dioguardi F, Mele D (2018). Pyflow 2.0: a computer program for calculating flow properties and impact paramters of past dilute pyroclastic density currents based on field data. Bull. Volcanol..

[65] Förster H, Oles D, Knittel U, Defant MJ, Torres RC (1990). The macolod corridor: a rift crossing the philippine island arc. Tectonophysics.

