# Supplementary material for: Hazardous base surges of Taal’s 2020 eruption
Source: Sci Rep. 2021 Aug 3;11:15703. doi: 10.1038/s41598-021-94866-2 (PMC8333357; doi:10.1038/s41598-021-94866-2)
Supplement: Supplementary file 1 — Supplementary material 1 (pdf 27 KB) [file 41598_2021_94866_MOESM1_ESM.pdf]

Legend for supplementary video
(file name: 13Jan2021_Vulcanian_Eruption)

Volcanic activity on 13 January 2020 characterized by a series of discrete, cannon-like explosions that were directed towards the west.
